# Experiences with Racism Among Asian American Medical Students

**DOI:** 10.1001/jamanetworkopen.2023.33067

**Published:** 2023-09-11

**Authors:** David H. Yang, Marissa Justen, Dana Lee, Heeryoung Kim, Dowin Boatright, Miraj Desai, Gunjan Tiyyagura

**Affiliations:** 1 Department of Emergency Medicine, Yale University School of Medicine, New Haven, Connecticut; 2 Yale University School of Medicine, Hew Haven, Connecticut; 3 Department of Psychiatry, Middlesex Hospital, Middletown, Connecticut; 4 Department of Emergency Medicine, New York University School of Medicine, New York; 5 Department of Psychiatry, Yale University School of Medicine, New Haven, Connecticut; 6 Department of Pediatrics and Emergency Medicine, Yale School of Medicine, New Haven, Connecticut

## Abstract

**Importance:**

Asian American physicians have experienced a dual pandemic of racism and COVID-19 since 2020; understanding how racism has affected the learning environment of Asian American medical students is necessary to inform strategies to promoting a more inclusive medical school environment and a diverse and inclusive workforce. While prior research has explored the influence of anti-Asian racism on the experiences of Asian American health care workers, to our knowledge there are no studies investigating how racism has impacted the training experiences of Asian American medical students.

**Objective:**

To characterize how Asian American medical students have experienced anti-Asian racism in a medical school learning environment.

**Design, Setting, and Participants:**

This qualitative study included online video interviews of Asian American medical students performed between July 29, 2021, and August 22, 2022. Eligible participants were recruited through the Asian Pacific American Medical Students Association and snowball sampling, and the sample represented a disaggregated population of Asian Americans and all 4 medical school years.

**Main Outcomes and Measures:**

The medical school experiences of Asian American medical students.

**Results:**

Among 25 participants, Asian ethnicities included 8 Chinese American (32%), 5 Korean American (20%), 5 Indian American (20%), 3 Vietnamese American (12%), 2 Filipino American (8%), and 1 (4%) each Nepalese, Pakistani, and Desi American; 16 (64%) were female. Participants described 5 major themes concerning their experience with discrimination: (1) invisibility as racial aggression (eg, “It took them the whole first year to be able to tell me apart from the other Asian guy”); (2) visibility and racial aggression (“It transitioned from these series of microaggressions that every Asian person felt to actual aggression”); (3) absence of the Asian American experience in medical school (“They’re not going to mention Asian Americans at all”); (4) ignored while seeking support (“I don’t know what it means to have this part of my identity supported”); and (5) envisioning the future.

**Conclusions and Relevance:**

In this qualitative study, Asian American medical students reported feeling invisible within medical school while a target of anti-Asian racism. Addressing these unique challenges related to anti-Asian racism is necessary to promote a more inclusive medical school learning environment.

## Key Points

**Question:**

How do Asian American medical students experience anti-Asian racism in medical school?

**Findings:**

This qualitative study of 25 Asian American medical students found that participants described 5 major themes: invisibility as racial aggression, visibility and racial aggression, absence of the Asian American experience in medical school, ignored while seeking support, and envisioning the future.

**Meaning:**

Results of this study suggest that Asian American medical students have not felt supported by their medical school during a period of heightened visibility due to anti-Asian racism, suggesting further attention is warranted by educators, medical schools, and institutions.

## Teaser

This qualitative study of interviews with Asian American medical students describes major themes of experiences with anti-Asian racism and discrimination.

## Introduction

Asian American health care workers (HCW) have experienced a rise in anti-Asian racism since the COVID-19 pandemic began. While the pandemic created a stressful environment for all health professionals,^1,2^ the concomitant rise of anti-Asian racism greatly threatened the well-being of Asian American HCWs.^3,4,5,6^ Exploring and addressing the impact of racism on the professional lives of Asian American HCWs is important in its own right and as part of a movement toward identifying barriers and promoting interventions that enhance workforce inclusion. Research among Asian American HCWs described a surge of racist experiences during the pandemic, spanning from racial microaggressions (defined as behavior or comments that subtly demean members of marginalized racial groups) to more blatant racism like racial profanities and derogatory racial stereotypes.^5,6,7,8^ Less is known about how racism has impacted the training experiences of Asian American medical students.

Medical school is a critical time for students to develop their foundation as physicians. Prior research has found that Asian American medical students experienced their medical school learning environment more negatively than their non-Hispanic White peers.^9^ Ahn et al^10^ described discrimination as an explanation for this differential learning environment. Similar to other racial and gender minoritized students, forms of discrimination range from disparate performance evaluations, reports of mistreatment and experiences of microaggressions, and honor society selection to the presence of racist stereotypes like the model minority, which supposes that all Asian Americans are successful and problem free.^9,10,11,12,13,14,15,16,17,18,19^ Since the COVID-19 pandemic began, several perspective pieces encouraged medical schools to actively combat anti-Asian racism.^20,21,22,23,24^ However, to our knowledge no research has reported on Asian American medical students experiences with anti-Asian racism during the pandemic.

Further investigation into Asian American medical students experiences is essential in developing evidence-based practices to enhance Asian American inclusion in medicine. In this rigorous qualitative study, we aimed to describe how Asian American medical students experience discrimination, investigate the impact of anti-Asian hate during the COVID-19 pandemic, and describe stakeholder-derived strategies that promote a more inclusive medical school environment.

## Methods

### Study Design

In this qualitative study, we performed 1-on-1 semistructured online video interviews to examine Asian American medical students experiences with discrimination. Participants provided verbal consent before participation. No financial compensation was provided to participants. The university’s institutional review board approved the study. The study followed the Consolidated Criteria for Reporting Qualitative Research (COREQ) reporting guideline.

Two interviewers (D.Y., H.K.) used an interview guide (eTable 1 in Supplement 1) consisting of open-ended questions and probes to encourage discussion. The interview guide was piloted with 2 Asian American medical students and iteratively revised throughout the interviews. Interviews lasted 37 to 79 minutes (median length, 58 minutes). Interviews were conducted via video calls (Zoom Video Communications) and were audiotaped and professionally transcribed. Data collection continued until we reached theoretical sufficiency.^25,26,27^ Our research team is described in eTable 2 in Supplement 1.

### Participants

Study investigators invited Asian American medical students to participate in the study through the Asian Pacific American Medical Students Association (APAMSA) listservs and on social media. Snowball sampling, in which existing participants recommended additional participants, was used to identify contacts who met inclusion criteria.^28^ One individual did not follow up to schedule an interview. Ethnicity was further clarified as participants self-identified during the interview. We use the term Asian American and not more inclusive terms like Asian American, Native Hawaiian, and Pacific Islander because no participants identified as Native Hawaiian or Pacific Islander.

### Data Analysis

Three authors (D.Y., M.J., D.L.) analyzed individual transcripts using the constant comparative method to identify a grounded theory model to describe the Asian American medical students experience with discrimination.^29^ First, we inductively identified initial codes outlining the experience of, response to, and reflection on experiences with racial microaggressions (open coding). The initial codebook was created by 1 interviewer (D.Y.) and 2 other members of the research team (M.J. and D.L.) with input from the 3 clinician researchers with extensive qualitative methods experience. Each transcript was initially coded independently by at least 2 research members as the codebook was developed and refined. All transcripts were independently recoded by 3 members of the research team once the codebook was finalized. Researchers examined associations within and between these codes (axial coding) to identify potentially useful ways to respond to microaggressions and eventually developed core themes (selective coding) that coalesced interview codes into a theoretical framework to explain the data. Additional input was provided by a phenomenological perspective, especially regarding the delineation and interrelation of core themes.^30,31,32,33^ To ensure dependability and credibility, we maintained an audit trail that included detailed memos on each team meeting. Investigator triangulation and member checking established the validity and trustworthiness of the data. NVivo version 12 (QSR International) was used for data storage, coding, and analysis.

## Results

Twenty-five Asian American medical students were interviewed. Asian ethnicities were 8 Chinese American (32%), 5 Korean American (20%), 5 Indian American (20%), 3 Vietnamese American (12%), 2 Filipino American (8%), and 1 (4%) each Nepalese, Pakistani, and Desi American; 16 (64%) were female (Table 1). Theoretical sufficiency was reached after 22 interviews. Medical students described 5 major themes concerning their experience with discrimination: (1) invisibility as racial microaggression: the experience of not being seen as an individual, but of being rendered as invisible because of race; (2) visibility and racial aggression: paradoxically, baseline experiences of being invisible were accompanied with experiences of being visible as a target of anti-Asian hate; (3) absence of the Asian American experience in medical school: teaching about Asian health issues was not part of the learning environment; (4) ignored while seeking support: Asian American medical students found limited support from medical school leadership during incidents of anti-Asian hate or in mental health support; and (5) envisioning the future: Asian American medical students recommended 4 key areas to create a more inclusive learning environment (Table 2).

**Table 1.** Participant Demographic Characteristics

Characteristic	Participants, No. (%) (N = 25)
Sex	
Male	9 (36)
Female	16 (64)
Medical school year	
First year	4 (16)
Second year	6 (24)
Third year	8 (32)
Fourth year	7 (28)
Self-identified ethnicity^a^	
Chinese American	8 (32)
Korean American	5 (20)
Indian American^a^	5 (20)
Nepalese American^a^	1 (4)
Pakistani American	1 (4)
Desi American^b^	1 (4)
Filipino American	2 (8)
Vietnamese American	3 (12)
Immigrant generation	
Born in the US	21 (84)
Born outside the US	4 (16)
Involvement with APAMSA	
Yes	23 (88)
No	3 (12)
Geographic location	
Midwest	7 (28)
Northeast	7 (28)
West	5 (20)
South	5 (20)
Other^c^	1 (4)

Abbreviation: APAMSA, Asian Pacific American Medical Students Association.

^a^ One participant identified as Indian and Nepalese American.

^b^ One participant self-identified as Desi American, which is a more inclusive term that includes many South Asian communities.

^c^ One participant went to a Caribbean medical school and was completing their rotations in the US at the time of interview.

**Table 2.** Themes and Representative Quotes

Theme and subtheme	Representative quotes
**Invisibility as racial aggression**	
Mistaken identity	“We’re supposed to have these mentors for small groups that we stick with for 4 years, and it took one of them the whole first year to be able to tell me apart from the other East Asian guy there.” Participant 23
Where are you from?	“All the time with patients where I try to put my foot down and say I’m from California and people don’t seem to accept [that].” Participant 14
Gendered discrimination	“You need to do 200 percent more than your [White] colleagues to be recognized and have people remember you.” Participant 14
	“‘She looks like Dr Pimple Popper…you’re a young, pretty Asian doctor like she is.’” Participant 17
**Visibility and racial aggression**	
Vicarious racism	“President Trump called it China virus—and the online, like, social media videos of elderly Asians getting beaten up—that was the first time I felt fear, not for myself, but for my mom and dad.” Participant 17
Carriers of disease, foreign enemies	“‘They might have the kung flu.’” Participant 11
**Absence of the Asian American experience in medical school**	
Admissions	“It’s sometimes frustrating to be grouped into this category of Asian American…specifically in regards to opportunities because he cannot apply it to a lot of the scholarships that he was hoping for because that is targeted towards minorities in medicine or in research.” Participant 9
Curriculum	“If you were to speak to a lot of different minority ethnic groups...Asian ethnic groups, you would sort of get the same sentiment in that Asians aren’t just Kawasaki disease or Takatsubo or whatever.” Participant 13
Student-run electives and minority tax	“A lot of this work is being done by people within minority communities and so that kind of puts the burden on us to be able to describe our experiences and talk about our own stories and leave ourselves vulnerable.” Participant 1
	“I felt guilty that I’m this student leader, like you’re not doing enough.…But then I also had to wear this hat of being a medical student, staying on top of things and having research projects going on and managing all of that.” Participant 7
Limited medical school acknowledgment in responding to Anti-Asian hate	“We did get an email from my school just wanting to address it [Anti-Asian rhetoric] and I think they basically would address all the misconceptions being said in the media and then throw in the blurb that the school counseling center is always there to talk to students.” Participant 12
Isolation and exhaustion	“They just don’t sit right with you and make you ruminate on them for the rest of the day.…It’s just it’s rough, it makes me really disheartened.” Participant 14
Barriers to seeking support	“When I was in my clerkship year, I was really lucky that my therapist had one or two slightly later evening slots cause clerkships run long. How do you get out in time to see someone?” Participant 20
	“If you experienced racial trauma is there a thing in place where you could take time to process? Do you have to go in and function as normal?” Participant 7
Areas of support	“APAMSA actually does have a faculty advisor...she was so helpful. I think she was like the main person like I reach out to. But other than that, I don’t know any other people.” Participant 7
	“As we came out of the room, the intern I was working with, she was like, ‘Are you okay? I just wanted to check in.’” Participant 21
**Envisioning the future**	
Education	“There’s a lot of Asian American minority groups here...I want the curriculum to kind of touch on that. How do we talk to Asian American or even to Asian immigrants in particular? How do we help them in a way that’s more supportive than right now, like focus on immigrant health and things like that. It’s not really covered in our curriculum so far.” Participant 7
Disaggregation	“I think disaggregating. The different populations that make up Asian would be also a good place to start.” Participant 8
Representation	“Seeing faculty and administration, people in positions of authority and power, look like me or look like my family members. That makes me feel like medicine is a space that I belong in and then I can feel safe in.” Participant 4
Allyship	“I think having my non-Asian peers talk about anti-Asian discrimination would be really great because it feels so good to feel seen for a long time.” Participant 4
	“She’s Asian American and I think she heard a racial slur from a patient that she was seeing. And her colleague, a nurse, African American nurse, said, you know, that was not right.” Participant 7

Abbreviation: APAMSA, Asian Pacific American Medical Students Association.

### Invisibility as Racial Microaggression

Medical students described a barrage of microaggressions that led to feelings of ostracization in the medical community. These microaggressions implied that they were invisible relative to other Asian American HCWs—“It took them the whole first year to be able to tell me apart from the other Asian guy.” One participant noted: “I’ve had attendings confuse me [for other Asian American students] before. I’ve had nurses and patients confuse me before.” This participant reported such microaggressions while working longitudinally with preceptors, and that the misidentifications continued despite attempts at correction. Asian American medical students worried about how this racialization would translate to their evaluations. One Chinese American medical student worried that he would receive another Asian American medical students’ evaluation because “[attendings] misattribute other things that other Asian Americans have said to me.”

Another common microaggression from classmates, patients, and other HCWs involved queries about where a student was from, which many participants interpreted as “you do not belong here.” An Indian American medical student described how she felt invisible except when seen as foreign: “all the time where I try to put my foot down and say I’m from California and people don’t seem to accept that.” Participants also noted how their experiences with discrimination often intersected with gender. Participants described being exoticized by patients: “You’re so pretty. You’re like a China doll,” observing that they “felt targeted, especially as an Asian woman.” Finally, a Pakistani American medical student discussed experiencing Islamophobia from his attending who told a patient that “He’s gonna get his buddies from the Taliban to come after you.”

### Visibility and Racial Aggression

Participants discussed numerous experiences of racial aggression because of the pandemic. Many participants described vicarious trauma from consuming media with depictions of elderly Asian Americans being physically assaulted—as one respondent said, “It transitioned from these series of microaggressions that every Asian person felt to actual aggression.” One Korean American medical student, reacting to a shooting in Atlanta where 6 Asian women were murdered in March 2021,^34^ described how the women who died “look like my mom.”

Asian American medical students also described being perceived as carriers of disease. A Filipino American medical student described how a patient’s parent yelled: “I don’t want that [Asian] nurse taking care of my child because I don’t want my kid to get coronavirus.” Other participants emphasized the contrast between working in health care and being Asian American: “I would scroll by a story that’s like someone (Asian) was assaulted and then the next story would be like…here are some signs thanking health care heroes. I was just like, well, which one am I going to get?”

### Absence of the Asian American Experience in Medical School

When discussing medical school admissions, participants expressed problems with being homogenized into “one group of Asians.” A respondent noted a moment of realization in medical school, “They’re not going to mention Asian Americans at all.” One participant described, “the Vietnamese community’s a good example where that migration history is so different [from] a lot of South Asian and East Asian migration histories…there’s a lot of active systemic exclusion in the context of class and race.” Another Chinese American medical student stressed the importance of including Asian ethnic minorities when creating pipelines to bring underrepresented students into medicine.

Most participants noted a complete absence of Asian American health curricula. One Chinese American medical student noted that “it’s harder to learn about the specific needs of Asian American patients.” A Korean American medical student described how, even in places with larger numbers of Asian Americans, there is no education: “there is a really high density of Japanese populations here in [city]…it would be nice to learn about how we can better serve that population.”

With this invisibility, student-run electives emerged as a space of presence—a space where Asian American individuals and perspectives were consistently acknowledged in medical school. Participants described APAMSA lectures on topics like the model minority myth and local Asian American health issues. However, participants worried about the minority tax—the extra obligation carried by individuals from minority groups to promote diversity in professional settings—that these student-run electives placed on Asian American medical students, describing that “a lot of this work is being done by people within minority communities and that puts the burden on us.”

### Ignored While Seeking Support

Given the pattern of invisibility for Asian American medical students, it follows that their struggles were invisible to the institutions around them. As one participant described it, part of the struggle was, “I don’t know what it means to have this part of my identity supported.” Their status as a visible target of post–COVID-19 or post-9/11 discrimination was often dismissed, leading to few institutional options for support seeking. Asian American hate crimes were often ignored by the medical school administration, leading students to feel unseen. A Vietnamese American medical student, describing her medical school’s response to the Atlanta shooting,^34^ expressed frustration: “I don’t think my school really issued any statement or did anything to address the issue.”

Many participants describe feeling isolated because their issues were invisible to others: “our Asian friends are checking in on each other but no one outside that racial group was checking in on us.” Others expressed feeling exhausted by discrimination: “I have been on the receiving end of microaggressions and comments that just don’t sit right with you and make you ruminate on them for the rest of the day.”

Despite frequent experiences with discrimination during COVID-19, Asian American medical students described barriers in seeking support at their medical school. One Chinese American medical student described her experience reaching out to an Asian American medical school dean: “I wish that I could help you with this, but I don’t know what to do that won’t put a target on your back more than it already is.” Students also struggled with their therapists: “faculty was White and the counselor was too, so sometimes it’s nice to talk to someone that may understand your background.”

Participants identified places and people outside of APAMSA who facilitated participants’ experience of visibility. One Chinese American medical student described how an Asian American attending supported him after a patient asked him “where are you from?” He described how she checked in with him without being overly imposing: “I realize it happened, and if you want to talk about it, I’m here.”

Feeling heard did not require that support comes from another Asian American. After an attending discussed how different races have different head shapes, one Korean American medical student described how a non-Asian resident supported her by text: “I think whatever he said was super inappropriate. And if you feel like you need a debrief, come talk to me.”

### Envisioning the Future

While describing the challenges faced by Asian American medical students, participants highlighted several interpersonal and institutional recommendations to enhance their training experience, sense of inclusion, and overall support. These broadly included: disaggregation, education, representation, and allyship.

Participants recommended that institutions disaggregate Asian American subpopulations during admissions and in the curriculum. Many felt that disaggregation of Asian subgroups would lead to additional support during the admission process for those that are disenfranchised. Disaggregation and recognition of issues affecting different Asian populations was also thought to lead to better health care for the Asian American population.

Education efforts were identified as critical to meeting the needs of Asian American medical students. Participants proposed that lectures focused on Asian American health be taught to all medical students, not just Asian American medical students. They preferred that lectures surrounding Asian American and Pacific Islander health be integrated into the medical curriculum to remove the burden of teaching from Asian American medical students. Increased focus on sociohistorical issues emerged as a clear and concrete need for health and medical training programs.

Participants identified the importance of increased representation of Asian Americans among faculty, administrators, and mental health personnel to combat the invisibility they experienced. Many medical students described how difficult it was to find someone they could connect with and how important it was to have Asian American supervisors with institutional power and support.

Allyship was highlighted as an essential way that non-Asian American individuals could support their Asian American colleagues. Examples of such allyship included bystander intervention and advocating for topics relevant to Asian Americans in lectures not taught by Asian Americans. Colleagues and medical school leaders who were present at educational events, helped organize these events, or simply named racism or discrimination were identified as particularly helpful.

## Discussion

Our study describes the changing landscape experienced by Asian American medical students during the COVID-19 pandemic because of racism. The microaggressions described in our study parallel experiences reported by other minoritized populations within medical school and residency.^35,36^ Participants described discrimination that included being seen as foreign enemies and carriers of disease. These findings highlight the “perpetual foreigner” perspective of Asian Americans, a phenomenon in which Asian Americans are more closely linked with an Asian country of origin rather than the US.^37,38^ Such findings echo previous studies that describe similar experiences by Asian American HCWs^5,6,8^ and South Asians after 9/11.^39,40,41,42^

Participants reported that these racialized experiences affected their mental health and made them feel invisible within school support structures, consistent with experiences described by other minoritized trainees in medicine^35,43,44^ and Asian American HCWs.^5,6,8,10,44,45^ These findings expand on a previous study that found that Asian American medical students felt excluded in conversations on diversity and inclusion^10^ and supports previous work that found institutional responses to microaggressions to be inadequate.^46^ Our findings suggest that further attention should be given to counter harmful stereotypes like the model minority stereotype and the perpetual foreigner stereotype. Our stakeholder-derived strategies from the final theme may offer guidance for this inclusion.

The testimony from these interviews suggests that the monolithic characterization of Asian American medical students further disenfranchises underrepresented Asian populations and is consistent with prior research demonstrating that grouping Asian Americans together make certain subpopulations invisible, which in turn translates to masking health disparities and mental health issues.^4,10,45,47,48,49^ The Association of American Medical Colleges has defined *underrepresentation* in medicine since 2003 as “racial and ethnic populations that are underrepresented in the medical profession relative to their numbers in the general population.”^50^ Disaggregation of medical school applicants based on this definition has shown that some subgroups, such as Bangladeshi, Laotian, Indonesian, and Cambodian populations, are underrepresented.^51^ No disaggregated data on matriculated Asian American medical students or other Asian American HCWs has been published thus far. Dissemination of workforce data on Asian American subgroups is advised, although this may not take nuances of within-country socioeconomic disparities into consideration.

Participants also described the absence of and desire for including Asian American health issues within the medical curriculum. This finding is consistent with previous work that found that Asian Americans were omitted and needed more dedicated time in the curriculum^52,53^ and expands prior studies by specifically calling for this teaching within the medical curriculum.^10^ These findings warrant the introduction of Asian American health and history lectures within the medical school curriculum.

We found that participants greatly benefited from the support resulting from having Asian American faculty and staff at their medical school. They reported a lack of Asian American representation and recommended increased representation at the faculty and leadership level. This finding is consistent with a body of research on limited representation of Asian American in leadership positions.^54,55,56^ Medical schools should work to increase Asian American representation in medical school leadership.

Finally, genuine allyship from peers, faculty, staff, and leadership provided another means of support to Asian American medical students. This finding is consistent with previous qualitative research where Asian Americans recommended training for the public to promote interventions by allies.^48^ Such sentiment is evidence based, as it has been shown that bystander intervention training is effective in addressing incidents of discrimination in a variety of education and clinical settings.^57,58,59,60^

### Limitations

This study has several limitations. First, our study reports perspectives of 25 students and is not meant to be representative of all Asian American medical students. For example, our study did not include the perspectives of many Asian ethnic populations like Hmong, Native Hawaiian, or Pacific Islander. Themes that emerged should be tested in future quantitative work to explore their influence on the Asian American medical students learning environment. Second, we conducted our study during the COVID-19 pandemic and during renewed attention to anti-Asian hate. Whether these events prompted sustained changes in medical school teaching requires further study. Third, most of our participants were involved with APAMSA. Their perspectives may differ from Asian American medical students who do not belong to this organization. Additionally, this study may be limited by social desirability bias. However, our interviewers ensured confidentiality and encouraged participants to provide honest responses.

## Conclusions

In this qualitative study, we found that Asian American medical students experience racism while in medical school, but remain invisible within the structure of medical school programs. While APAMSA, a few Asian American medical leaders, and some allies are notable exceptions, support for this population is limited and has not met its needs during the rise of anti-Asian racism since the COVID-19 pandemic began. Medical schools should pursue individual-level and institutional reform, with particular attention to addressing the invisibility felt by Asian American medical students.

## Notes

Supplement 1. **eTable 1.** Interview Guide **eTable 2.** Research Team and Reflexivity

Supplement 2.
Data Sharing Statement

## References

[1] Christophers B, Nieblas-Bedolla E, Gordon-Elliott JS, Kang Y, Holcomb K, Frey MK. Mental health of US medical students during the COVID-19 pandemic. J Gen Intern Med. 2021;36(10):3295-3297. doi:10.1007/s11606-021-07059-y 34355345 PMC8341832

[2] Jaconia GD, Lynch LR, Miller LK, Hines RL, Pinyavat T. COVID-19 impact on resident mental health and well-being. J Neurosurg Anesthesiol. 2022;34(1):122-126. doi:10.1097/ANA.0000000000000806 34870634

[3] Escobedo LA, Morey BN, Sabado-Liwag MD, Ponce NA. Lost on the frontline, and lost in the data: COVID-19 deaths among Filipinx healthcare workers in the United States. Front Public Health. 2022;10:958530. doi:10.3389/fpubh.2022.958530 36091528 PMC9452815

[4] Kalyanaraman Marcello R, Dolle J, Tariq A, . Disaggregating Asian race reveals COVID-19 disparities among Asian American patients at New York City’s public hospital system. Public Health Rep. 2022;137(2):317-325. doi:10.1177/00333549211061313 34965776 PMC8900247

[5] Sangalang CC. “I’m sick of being called a hero—I want to get paid like one”: Filipino American frontline workers’ health under conditions of COVID-19 and racial capitalism. Front Public Health. 2022;10:977955. doi:10.3389/fpubh.2022.977955 36504981 PMC9726904

[6] Shang Z, Kim JY, Cheng SO. Discrimination experienced by Asian Canadian and Asian American health care workers during the COVID-19 pandemic: a qualitative study. CMAJ Open. 2021;9(4):E998-E1004. doi:10.9778/cmajo.20210090 34785529 PMC8598237

[7] Sue DW, Capodilupo CM, Torino GC, . Racial microaggressions in everyday life: implications for clinical practice. Am Psychol. 2007;62(4):271-286. doi:10.1037/0003-066X.62.4.271 17516773

[8] Kim SS, Roberts TME, Khusbo JE, Watriboonruang W, Parks A, Lewczyk J. Asian American nursing students’ experiences of racial microaggressions amid the COVID-19 pandemic: focus group discussions. Nurse Educ Pract. 2022;64:103459. doi:10.1016/j.nepr.2022.103459 36183568 PMC9501614

[9] Hill KA, Samuels EA, Gross CP, . Assessment of the prevalence of medical student mistreatment by sex, race/ethnicity, and sexual orientation. JAMA Intern Med. 2020;180(5):653-665. doi:10.1001/jamainternmed.2020.0030 32091540 PMC7042809

[10] Ahn DJ, Garg N, Naik AG, . Where do I fit in? A perspective on challenges faced by Asian American medical students. Health Equity. 2021;5(1):324-328. doi:10.1089/heq.2020.0158 34036216 PMC8140354

[11] Boatright D, Ross D, O’Connor P, Moore E, Nunez-Smith M. Racial disparities in medical student membership in the Alpha Omega Alpha Honor Society. JAMA Intern Med. 2017;177(5):659-665. doi:10.1001/jamainternmed.2016.9623 28264091 PMC5818775

[12] Dyrbye LN, Thomas MR, Eacker A, . Race, ethnicity, and medical student well-being in the United States. Arch Intern Med. 2007;167(19):2103-2109. doi:10.1001/archinte.167.19.2103 17954805

[13] Lee S, Juon HS, Martinez G, . Model minority at risk: expressed needs of mental health by Asian American young adults. J Community Health. 2009;34(2):144-152. doi:10.1007/s10900-008-9137-1 18931893 PMC3296234

[14] Nguyen M, Chaudhry SI, Desai MM, . Association of mistreatment and discrimination with medical school attrition. JAMA Pediatr. 2022;176(9):935-937. doi:10.1001/jamapediatrics.2022.1637 35639402 PMC9157380

[15] Nguyen M, Song SH, Ferritto A, Ata A, Mason HRC. Demographic factors and academic outcomes associated with taking a leave of absence from medical school. JAMA Netw Open. 2021;4(1):e2033570. doi:10.1001/jamanetworkopen.2020.33570 33481029 PMC7823220

[16] Wear D. Asian/Pacific Islander women in medical education: personal and professional challenges. Teach Learn Med. 2000;12(3):156-163. doi:10.1207/S15328015TLM1203_7 11228903

[17] Zhang L, Lee ES, Kenworthy CA, . Southeast and East Asian American medical students’ perceptions of careers in academic medicine. J Career Dev. 2019;46(3):235-250. doi:10.1177/0894845317740225

[18] Hill KA, Desai MM, Chaudhry SI, . Association of marginalized identities with Alpha Omega Alpha Honor Society and Gold Humanism Honor Society membership among medical students. JAMA Netw Open. 2022;5(9):e2229062. doi:10.1001/jamanetworkopen.2022.29062 36069984 PMC9453541

[19] Boatright D, Anderson N, Kim JG, . Racial and ethnic differences in internal medicine residency assessments. JAMA Netw Open. 2022;5(12):e2247649. doi:10.1001/jamanetworkopen.2022.47649 36580337 PMC9857126

[20] Chang RS, Hu J-R, Kagetsu NJ. Acknowledge racism in academic medicine: addressing and preventing attacks on Asian Americans and Pacific Islanders. Acad Med. 2022;97(6):766-767. doi:10.1097/ACM.0000000000004647 35703904

[21] Liu CZ, Wang E, Nguyen D, Sun MD, Jumreornvong O. The model minority myth, data aggregation, and the role of medical schools in combating anti-Asian sentiment. Acad Med. 2022;97(6):797-803. doi:10.1097/ACM.0000000000004639 35703909

[22] Ko M, Ton H. The not underrepresented minorities: Asian Americans, diversity, and admissions. Acad Med. 2020;95(2):184-189. doi:10.1097/ACM.0000000000003019 31577586

[23] Wang MZ, Hu JR. Asian American trainees during the COVID-19 pandemic. Acad Med. 2021;96(6):780. doi:10.1097/ACM.0000000000004038 33656002 PMC8140622

[24] Wang V, Wang MJ, Lee YW. Reflections on the Asian American and Pacific Islander experience: not a monolith—a movement. Obstet Gynecol. 2021;138(2):284-288. doi:10.1097/AOG.0000000000004472 34237758

[25] Hanson JL, Balmer DF, Giardino AP. Qualitative research methods for medical educators. Acad Pediatr. 2011;11(5):375-386. doi:10.1016/j.acap.2011.05.001 21783450

[26] LaDonna KA, Artino AR Jr, Balmer DF. Beyond the guise of saturation: rigor and qualitative interview data. J Grad Med Educ. 2021;13(5):607-611. doi:10.4300/JGME-D-21-00752.1 34721785 PMC8527935

[27] Varpio L, Ajjawi R, Monrouxe LV, O’Brien BC, Rees CE. Shedding the cobra effect: problematising thematic emergence, triangulation, saturation and member checking. Med Educ. 2017;51(1):40-50. doi:10.1111/medu.13124 27981658

[28] Curry LA, Nembhard IM, Bradley EH. Qualitative and mixed methods provide unique contributions to outcomes research. Circulation. 2009;119(10):1442-1452. doi:10.1161/CIRCULATIONAHA.107.742775 19289649

[29] Strauss AL, Corbin JM. Basics of Qualitative Research: Grounded Theory Procedures and Techniques. Sage Publications; 1990.

[30] Wertz FJ. Phenomenological research methods for counseling psychology. J Couns Psychol. 2005;52:167-177. doi:10.1037/0022-0167.52.2.167

[31] Wertz FJ. The method of eidetic analysis for psychology. In: Cloonan TF, Thiboutot C, eds. The Redirection of Psychology: Essays in Honor of Amedeo P. Giorgi. Le Cercle Interdisciplinaire de Recherches Phénoménologiques; 2010:261-278.

[32] Desai MU, Guy K, Brown M, . “That was a state of depression by itself dealing with society”: atmospheric racism, mental health, and the Black and African American faith community. Am J Community Psychol. 2023. doi:10.1002/ajcp.12654 37006193 PMC10545810

[33] Desai MU, Paranamana N, Dovidio JF, Davidson L, Stanhope V. System-centered care: how bureaucracy and racialization decenter attempts at person-centered mental health care. Clin Psychol Sci. 2023;11(3):476-489. doi:10.1177/21677026221133053 37333799 PMC10275339

[34] Chen M. ‘She could have been your mother’: anti-Asian racism a year after Atlanta spa shootings. *The Guardian*. Published March 16, 2022. Accessed March 5, 2023. https://www.theguardian.com/us-news/2022/mar/16/anti-asian-racism-atlanta-spa-shootings-anniversary

[35] Osseo-Asare A, Balasuriya L, Huot SJ, . Minority resident physicians’ views on the role of race/ethnicity in their training experiences in the workplace. JAMA Netw Open. 2018;1(5):e182723. doi:10.1001/jamanetworkopen.2018.2723 30646179 PMC6324489

[36] de Bourmont SS, Burra A, Nouri SS, . Resident physician experiences with and responses to biased patients. JAMA Netw Open. 2020;3(11):e2021769. doi:10.1001/jamanetworkopen.2020.21769 33226429 PMC7684448

[37] Daley JS, Gallagher NM, Bodenhausen GV. The pandemic and the “perpetual foreigner”: how threats posed by the COVID-19 pandemic relate to stereotyping of Asian Americans. Front Psychol. 2022;13:821891. doi:10.3389/fpsyg.2022.821891 35250760 PMC8895344

[38] Kim SY, Wang Y, Deng S, Alvarez R, Li J. Accent, perpetual foreigner stereotype, and perceived discrimination as indirect links between English proficiency and depressive symptoms in Chinese American adolescents. Dev Psychol. 2011;47(1):289-301. doi:10.1037/a0020712 21244164 PMC3052861

[39] Iwamoto DK, Negi NJ, Partiali RN, Creswell JW. The racial and ethnic identity formation process of second-generation Asian Indian Americans: a phenomenological study. J Multicult Couns Dev. 2013;41(4):224-239. doi:10.1002/j.2161-1912.2013.00038.x 25298617 PMC4186661

[40] Inman AG, Altman A, Kaduvettoor-Davidson A, Carr A, Walker JA. Cultural intersections: a qualitative inquiry into the experience of Asian Indian-White interracial couples. Fam Process. 2011;50(2):248-266. doi:10.1111/j.1545-5300.2011.01358.x 21564064

[41] Livengood JS, Stodolska M. The effects of discrimination and constraints negotiation on leisure behavior of American Muslims in the post-September 11 America. J Leis Res. 2004;36(2):183-208. doi:10.1080/00222216.2004.11950019

[42] Unni AK, Blake JJ, Salter PS, Luo W, Liew J. “No, but where are you *really* from?” Experiences of perceived discrimination and identity development among Asian Indian adolescents. Front Public Health. 2022;10:955011. doi:10.3389/fpubh.2022.955011 36330127 PMC9623414

[43] Ackerman-Barger K, Boatright D, Gonzalez-Colaso R, Orozco R, Latimore D. Seeking inclusion excellence: understanding racial microaggressions as experienced by underrepresented medical and nursing students. Acad Med. 2020;95(5):758-763. doi:10.1097/ACM.0000000000003077 31725462 PMC7185051

[44] Pryce-Miller M, Bliss E, Airey A, Garvey A, Pennington CR. The lived experiences of racial bias for Black, Asian and Minority Ethnic students in practice: a hermeneutic phenomenological study. Nurse Educ Pract. 2023;66:103532. doi:10.1016/j.nepr.2022.103532 36563599

[45] Yang D, Oral E, Kim J, Craft T, Moore MB. Depression and perceived social support in Asian American medical students. J Racial Ethn Health Disparities. 2021. 33876407 10.1007/s40615-021-01043-2

[46] Anderson N, Lett E, Asabor EN, . Depression and perceived social support in Asian American medical students. J Racial Ethn Health Disparities. 2022;9(3):1040-1050. doi:10.1007/s40615-021-01043-2 33876407

[47] Chang B, Au W. You’re Asian, how could you fail at math? Unmasking the myth of the model minority. In: Rethinking Multicultural Education: Teaching for Racial and Cultural Justice. Au W, ed. Rethinking Schools; 2009:207-216.

[48] Wang SC, Santos BMC. “What support?”: A qualitative study on social support for Asian American victims of racism during the COVID-19 pandemic. Front Public Health. 2022;10:961215. doi:10.3389/fpubh.2022.961215 36339164 PMC9626990

[49] Young JL, Cho MK. The invisibility of Asian Americans in COVID-19 data, reporting, and relief. Am J Bioeth. 2021;21(3):100-102. doi:10.1080/15265161.2020.1870767 33616487 PMC11040541

[50] Association of American Medical Colleges. Underrepresented in Medicine Definition. Accessed June 5, 2023. https://www.aamc.org/what-we-do/equity-diversity-inclusion/underrepresented-in-medicine

[51] Association of American Medical Colleges. Diversity in Medicine: Facts and Figures 2019. Published March 20, 2019. Accessed December 1, 2022. https://www.aamc.org/data-reports/workforce/interactive-data/figure-3-percentage-asian-alone-applicants-us-medical-schools-asian-subgroups-academic-year-2018

[52] Park PSU, Algur E, Narayan S, Song WB, Kearney MD, Aysola J. Representation of Asian American populations in medical school curricula. JAMA Netw Open. 2022;5(9):e2233080. doi:10.1001/jamanetworkopen.2022.33080 36149654 PMC9508660

[53] Le TK, Vongsachang H, Pang S, . US medical student perspectives on Asian American patient inclusion in medical education: a qualitative study. BMC Med Educ. 2022;22(1):482. doi:10.1186/s12909-022-03550-0 35729562 PMC9213094

[54] Choi AMK, Rustgi AK. Diversity in leadership at academic medical centers: addressing underrepresentation among Asian American faculty. JAMA. 2021;326(7):605-606. doi:10.1001/jama.2021.12028 34402820

[55] Yu PT, Parsa PV, Hassanein O, Rogers SO, Chang DC. Minorities struggle to advance in academic medicine: a 12-y review of diversity at the highest levels of America’s teaching institutions. J Surg Res. 2013;182(2):212-218. doi:10.1016/j.jss.2012.06.049 23582226

[56] Gee B, Peck D. Asian Americans are the least likely group in the US to be promoted to management. Harvard Business Review. Published May 31, 2018. Accessed March 1, 2023. https://hbr.org/2018/05/asian-americans-are-the-least-likely-group-in-the-u-s-to-be-promoted-to-management

[57] Ackerman-Barger K, Jacobs NN, Orozco R, London M. Addressing microaggressions in academic health: a workshop for inclusive excellence. MedEdPORTAL. 2021;17:11103. doi:10.15766/mep_2374-8265.11103 33598543 PMC7880252

[58] Coker AL, Bush HM, Cook-Craig PG, . RCT testing bystander effectiveness to reduce violence. Am J Prev Med. 2017;52(5):566-578. doi:10.1016/j.amepre.2017.01.020 28279546 PMC5737001

[59] Famouri ML, Hernandez S, Omlor RL, . Active bystander training: using standardized patient methodology to teach residents to navigate microaggressions in patient encounters. MedEdPORTAL. 2023;19:11298. doi:10.15766/mep_2374-8265.11298 36760336 PMC9886691

[60] York M, Langford K, Davidson M, . Becoming active bystanders and advocates: teaching medical students to respond to bias in the clinical setting. MedEdPORTAL. 2021;17:11175. doi:10.15766/mep_2374-8265.11175 34485695 PMC8374028

