# Supplementary material for: Experiences with Racism Among Asian American Medical Students
Source: JAMA Netw Open. 2023 Sep 11;6(9):e2333067. doi: 10.1001/jamanetworkopen.2023.33067 (PMC10495868; doi:10.1001/jamanetworkopen.2023.33067)
Supplement: Supplement 1. — eTable 1. Interview Guide eTable 2. Research Team and Reflexivity [file jamanetwopen-e2333067-s001.pdf]

# Supplemental Online Content

Yang DH, Justen M, Lee D, et al. Experiences with racism among Asian American medical students. *JAMA Netw Open.* 2023;6(9):e2333067. doi:10.1001/jamanetworkopen.2023.33067

**eTable 1.** Interview Guide

**eTable 2.** Research Team and Reflexivity

This supplemental material has been provided by the authors to give readers additional information about their work.

© 2023 Yang DH et al. *JAMA Network Open.*

## eTable1. Interview Guide

Good afternoon! Thank you for joining me/us for this interview. As you know, the purpose of this study is to contribute Asian American medical student perspectives to the body of knowledge related to promoting student success in schools of medicine. In particular, we'll be focusing on discrimination and racial microaggressions during the pandemic. Greater understanding of how they are experienced can lead to increased support for students to help them reach their full academic potential.

1. Can you introduce yourself and tell me a little bit about yourself?
    a. Tell me about how your identity has impacted your experiences during the pandemic.
2. What sources of support do you have in medical school?
    a. How has the pandemic impacted these sources of support?
    b. How have you felt supported during this pandemic?
    c. How have you supported yourself during this pandemic?
3. How does your identity as an "Asian American" influence your experiences during the COVID-19 pandemic?
4. Imagine yourself before COVID19 started. Had you (or others close to you) experienced or witnessed any forms of microaggressions or discrimination related to being Asian American? If so, would you describe the circumstances?
5. Have you (or others close to you) experienced or witnessed any forms of microaggressions or discrimination related to being Asian American since the pandemic began? If so, would you describe the circumstances?
6. Tell me about your experience with discrimination in the medical environment.
7. In the context of discussing discrimination as Asian American in medical school, what can a medical school do to create an ideal learning environment?
8. As you know, when we first started talking, I asked you about supports you have in medical school. What supports could be added to create an ideal learning environment?

© 2023 Yang DH et al. *JAMA Network Open.*

**eTable2. Research Team and Reflexivity**

| |
|---|
| Interviews were facilitated by study authors (D.Y. and H.K.), who identify as a Chinese American male emergency resident physician and a Korean American female psychiatry resident physician. Interviewers were trained in qualitative research and did not have pre-existing connections to participants that they interviewed. The first author (D.Y.) was also involved in coding. The other two coders (M.J., and D.L.) self-identified as an East Asian American medical student, and a Chinese American medical student. The three clinician researchers with extensive experience in qualitative methods (D.B., M.D., and G.T.) guided the coding and analysis process and self-identified as an African American male emergency physician-researcher, a South Asian American female pediatric emergency physician-researcher, and a South Asian American male psychologist-researcher. |

© 2023 Yang DH et al. *JAMA Network Open.*
