# Supplementary material for: Experiences with Racism Among Asian American Medical Students
Source: JAMA Netw Open. 2023 Sep 11;6(9):e2333067. doi: 10.1001/jamanetworkopen.2023.33067 (PMC10495868; doi:10.1001/jamanetworkopen.2023.33067)
Supplement: Supplement 2. — Data Sharing Statement [file jamanetwopen-e2333067-s002.pdf]

Data Sharing Statement

Yang. Experiences with Racism Among Asian American Medical Students. *JAMA Netw Open*. Published September 11, 2023. doi:10.1001/jamanetworkopen.2023.33067

Data
Data available: No
